# Growth, physiological, and biochemical responses of *Camptotheca acuminata* seedlings to different light environments

**DOI:** 10.3389/fpls.2015.00321

**Published:** 2015-05-08

**Authors:** Xiaohua Ma, Lili Song, Weiwu Yu, Yuanyuan Hu, Yang Liu, Jiasheng Wu, Yeqing Ying

**Affiliations:** ^1^Nurturing Station for the State Key Laboratory of Subtropical Silviculture, Zhejiang A & F UniversityHangzhou, China; ^2^School of Forestry and Biotechnology, Zhejiang A & F UniversityHangzhou, China

**Keywords:** *Camptotheca acuminata*, light intensity, photosynthetic characteristics, chlorophyll fluorescence, antioxidant enzyme activity, chloroplast ultrastructure

## Abstract

Light intensity critically affects plant growth. *Camptotheca acuminata* is a light-demanding species, but its optimum light intensity is not known. To investigate the response of *C. acuminata* seedlings to different light intensities, specifically 100% irradiance (PAR, 1500 ± 30 μmol m^−2^ s^−1^), 75% irradiance, 50% irradiance, and 25% irradiance, a pot experiment was conducted to analyze growth parameters, photosynthetic pigments, gas exchange, chlorophyll fluorescence, stomatal structure and density, chloroplast ultrastructure, ROS concentrations, and antioxidant activities. Plants grown under 75% irradiance had significantly higher total biomass, seedling height, ground diameter, photosynthetic capacity, photochemical efficiency, and photochemical quenching than those grown under 100%, 25%, and 50% irradiance. Malondialdehyde (MDA) content, relative electrolyte conductivity (REC), superoxide anion (O^.−^_2_) production, and peroxide (H_2_O_2_) content were lower under 75% irradiance. The less pronounced plant growth under 100% and 25% irradiance was associated with a decline in photosynthetic capacity and photochemical efficiency, with increases in the activity of specific antioxidants (i.e., superoxidase dismutase, peroxidase, and catalase), and with increases in MDA content and REC. Lower levels of irradiance were associated with significantly higher concentrations of chlorophyll (Chl) a and b and lower Chla/b ratios. Stomatal development was most pronounced under 75% irradiance. Modification of chloroplast development was found to be an important mechanism of responding to different light intensities in *C. acuminata*. The results indicated that 75% irradiance is optimal for the growth of *C. acuminata* seedlings. The improvement in *C. acuminata* growth under 75% irradiance was attributable to increased photosynthesis, less accumulation of ROS, and the maintenance of the stomatal and chloroplast structure.

## Introduction

*Camptotheca acuminata* Decne., which belongs to the Nyssaceae family, is a deciduous hardwood native to China. It has been used extensively for ornamental and medicinal purposes. Recently, *C. acuminata* has become increasingly important because of the high content of camptothecins (CPT) which are natural secondary metabolites and have shown marked effects in anti-tumor, immune deficiency disease resistance in various parts of it (Li et al., 2002). Despite CPT has been isolated from a variety of species of families of angiosperms, such as *Nothapodytes foetida* (Aiyama et al., 1988) and *Pyrenacantha klaineana* (Zhou et al., 2000), the highest level of CPT was found in young leaves at juvenile stages in *C. acuminata* which has been used for commercial CPT production (López-Meyer et al., 1994). Large-scale leaf-producing plantation of *C. acuminata* has been established for CPT production, which not only depends on leaf biomass production but also concentration of CPT in leaf. It has been reported that stresses such as light and drought before leaf harvest could increase CPT concentration in *C. acuminata* leaves, while such stresses would inhibit the growth of *C. acuminate* and decrease the accumulation of leaves biomass (Liu et al., 1997, 2015; Liu, 2000). Therefore, it is necessary to determine the optimum environment factors for better growth of *C. acuminata* and high leaf biomass production.

Light is one of the most important requirements for plant growth and the effects of changes in light intensity on plant growth, morphology photosynthetic capacity, various aspects of physiology and biochemistry, and ultimately productivity, are well-known (Dai et al., 2009). Typically, normal plant growth requires optimal light irradiance and excessive high or low irradiance impacts photosynthesis, which is central to plant productivity, and can therefore severely restrict plant growth. For example, Powles and Critchley (1980) reported bean plants grown under low light had lower rates of photosynthetic electron transport and carbon dioxide (CO_2_) assimilation than leaves of plants grown in full sunlight, leading to reduced growth in *Tabebuia chrysotricha*. However, exposure of bayberry tree to a high irradiance (1300 μmol m^−2^ s^−1^) can cause a depression of photosynthesis and photosystem II (PSII) efficiency (Guo et al., 2006). These responses can result in photoinhibition caused by excessive light energy, and/or impairment of the chlorophyll-containing reaction centers of the chloroplasts and a significant reduction in the electron transport rate (ETR) and photochemical quenching (qP), and an increase in non-photochemical quenching (NPQ) (Bertaminia et al., 2006). Many protective mechanisms from photoinhibiton involve processes such as chlorophyll (Chl) content change (Murchie and Horton, 1997), chloroplast movement, increase the capacity for scavenging the active oxygen species by means of increase in scavenging enzyme activity and/or concentration of non-enzymatic antioxidants (Foyer et al., 1994). High levels of POD, SOD, and CAT enzyme activity which enable the rapid clearance of ^.−^O_2_ and catalyze the decomposition of H_2_O_2_ to water and oxygen were found to be induced by high irradiance in olive trees (Sofo et al., 2004).

*C. acuminata* is considered a light-demanding species. It is planted mainly in southern China where there is sufficient light intensity for growth. During the artificial establishment of *C. acuminata*, the survival, early growth, and leaf biomass production of these tree seedlings are strongly affected by light intensity (Feng et al., 2008). In particular, high light irradiance during the hot summer season can injure *C. acuminata* seedlings. Knowledge of the morphological and physiological characteristics of *C. acuminata* in response to various light conditions is still sparse. In the present study, the growth, photosynthetic characteristics, antioxidant defense systems, and ultrastructure of the stoma and chloroplast were investigated in *C. acuminata* seedlings grown under various light levels to determine the optimal light conditions for this species and to assess acclimation under different light conditions to provide information for improved cultivation. This study was designed to address two issues: (1) the optimum light intensity for *C. acuminata* growth; (2) reaction of morphology, photosynthesis, and antioxidant defense of *C. acuminata* seedlings to various light conditions. It is anticipated that this information will contribute to expansion of our understanding of the light-regulating mechanism in this species to provide a sound theoretical foundation for the standardized cultivation of this important medicinal plant.

## Materials and methods

### Plant materials and growth conditions

**Figure 1:**
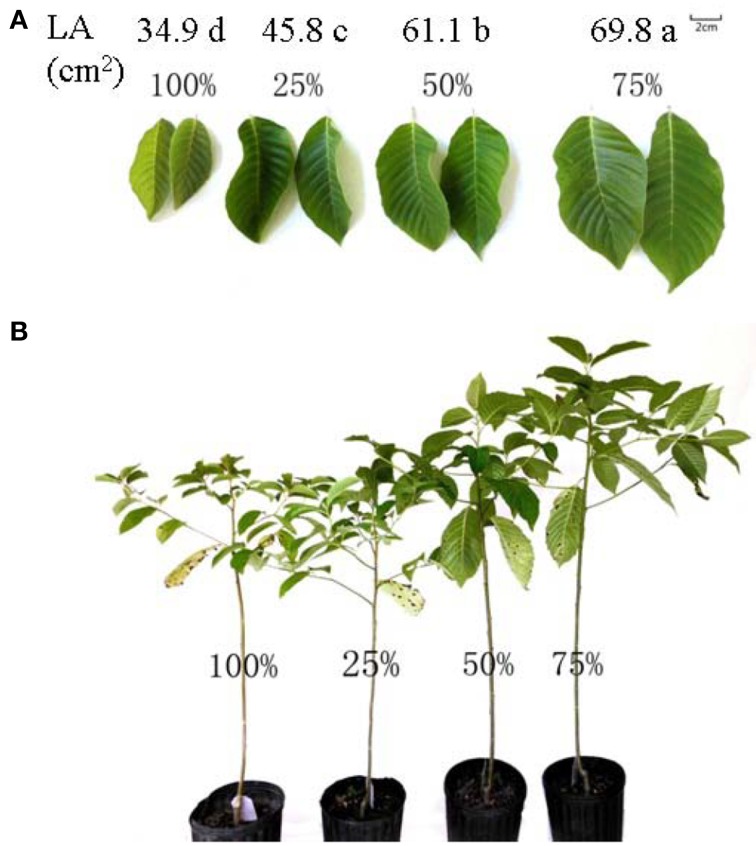
***C. acuminata* leaves and leaf area (A) and seedlings (B)**. 100% level = 1500 ± 30 μmol m^−2^ s^−1^.

In late March 2013, a pot experiment was established to examine the effects of different light intensities on the physiological and biochemical changes in *C. acuminata* seedlings. The experiment was conducted with different thickness shading nets in a room with a controlled environment at Zhejiang A&F University (30°23′N, 119°72′E) in China. One-year-old healthy and homogenous *C. acuminata* seedlings (mean ground diameter 5.8 ± 5 mm and seedling height 42.4 ± 2 cm) were transferred to plastic pots (16.5 cm inner diameter, 18 cm height, with holes in the bottom, one seedling per pot) filled with a substrate mixture of pine bark: peat: soil (4:4:2, v/v/v, 40 kg m^−3^ of organic manure). All the pots were irrigated daily to keep the plants well-watered (the water level was kept at 75% of the field capacity of the soil). After 8 weeks of growth, 60 of uniform seedlings were divided into four groups. A completely randomized design with five replications per treatment and three plants per replication was set up. The seedlings per replication were moved into the growth chamber under artificial light (six 400 W dysprosium lamps above 10-cm water layer serving as heat filter). Photosynthetically active radiation (PAR, 1500 ± 30 μmol m^−2^ s^−1^) was provided by adjusting the distance of the lamps to the plant canopies at 15 cm. Four irradiance levels were created by neutral shadecloths which have a neutral effect on light quality (Yates, 1989) over rigid frame, namely 100% irradiance (non-shaded, control), 25% irradiance (75% shaded), 50% irradiance (50% shaded), and 75% irradiance (25% shaded). All treatments were kept at 30 ± 2°C and 50–60% relative humidity during the day, 20 ± 2°C at night and 50–60% relative humidity with a 8–10 h light: 14–16 h dark photoperiod. The light intensity was measured with a Digital Lux Meter (TES-1339R, Taiwan). The plants were kept well-watered once a day until the end of the experiment. After 60 days of treatment, obvious external differences of plants under different light intensity treatments could be visually observed (Figure 1). Materials for the measurements of photosynthetic rate, antioxidant enzymes, lipid peroxidation, membrane permeability, chlorophyll content, superoxide anion production rate and the peroxide content were collected from leaves (the third and the fourth from the top) of five replicates per treatment, cleaned with tissue papers to remove any surface contamination and immediately frozen in liquid nitrogen and stored at −70°C.

### Plant growth and biomass

At the end of experiment (60 days), plant height was measured from the base of the main stem from the soil surface to the tip of the uppermost shoot using a ruler, and the ground diameter was measured at the internode 1-cm above the ground with vernier calipers; these measurements were conducted on each seedling in all treatments. Roots were elutriated with water to remove soil. One intact plant (above-ground shoot and below-ground root) from five replicates per treatment was harvested for biomass determination. The dry mass, including roots, stems, and leaves was obtained after oven drying at 80°C until a constant mass. The leaf area (LA) was measured with a LA meter (LI-300, Li-Cor, Lincon, NE, USA). Leaf mass ratio (LMR) was calculated by leaf dry mass divided by total biomass for each plant.

### Photosynthetic parameters

The youngest, healthy, fully-developed leaves (the third and the fourth from the top) from one plant randomly selected from one replication per treatment were used for photosynthetic measurements. Photosynthesis (Pn) was measured with a LI-6400 portable photosynthesis system (Li-Cor, Inc., Lincoln, NE, U.S.) with a standard leaf chamber equipped with a 6400-02B LED light source. Data were recorded between 9:30 and 11:30 a.m. at an air concentration of 21% O_2_, 400 μmol mol^−1^ CO_2_, 1200 μmol m^−2^ s^−1^ PAR white light and 60% relative humidity and a temperature of 30°C. Leaf and air temperature and relative humidity were used to calculate vapor pressure deficit between leaf and air (VPD). In the experiment, VPD was similar among the four treatments.

### Photosynthetic pigments

After the measurement of photosynthesis, approximately 0.1 g of a finely cut and well-mixed leaf samples from one plant randomly selected from one replication per treatment were extracted with 8 mL of 95% acetone. Chlorophyll was extracted at 4°C for 24 h in the dark and shaken three or four times until the samples were blanched. The absorbance was measured at 646, 663, and 450 nm with a spectrophotometer (Shimadzu UV-2550, Kyoto, Japan) after centrifugation the mixture. Chlorophyll concentrations were calculated with a standard method (Arnon, 1949) and expressed as mg g^−1^ fresh weight (FW).

### Chlorophyll fluorescence

Chlorophyll fluorescence was measured using a portable pulse modulation fluorometer (PAM 2500, Waltz GmbH, Effeltrich, Germany). Leaves from one plant randomly selected from one replication per treatment were dark-adapted for approximately 10 min (based on the previous experiment) and then initial fluorescence (Fo), maximal fluorescence (Fm), actual photochemical efficiency of PSII (Φ PSII), photochemical quenching (qP), and non-photochemical quenching (NPQ) were measured at 1200 μmol m^−2^ s^−1^ PAR. After the removal of the actinic light source and application of 3 s of far-red light, the minimal fluorescence of the light-adapted state (Fo') was obtained. Steady state fluorescence (Fs) was determined under actinic light (λ = 665 nm). The relative effective quantum yield of photochemical energy conversion at steady-state photosynthesis was calculated as Yield = (Fm' − Fs)/Fm', where Fs and Fm' are the fluorescence at steady-state photosynthesis and maximum fluorescence in the light, respectively. qP, Φ PSII, and NPQ were calculated as (Fm' − Fs)/(Fm' − Fo'), (Fm' − Fs)/Fm', and (Fm − Fm')/Fm, respectively (Genty et al., 1989).

### Determination of lipid peroxidation and membrane permeability

The lipid peroxidation level was determined in terms of the malondialdehyde (MDA) content using a method described by Deng et al. (2012). Leaves from one plant randomly selected from one replication per treatment (1.0 g) were ground in 10% trichloroacetic acid and centrifuged at 3000 × *g* for 10 min. Then 2 mL of 0.6% thiobarbituric acid in 10% TCA was added to each 2 mL of aliquot of the supernatant. The mixtures were heated at 100°C for 30 min and then rapidly cooled in an ice bath. After centrifugation at 5000 × *g* for 20 min, the absorbance of the supernatant was recorded at 532, 600, and 450 nm. Lipid peroxidation was expressed as μmol g^−1^ using the following formula: 6.45(A_532_ − A_600_) − 0.56A_450_.

Membrane permeability was estimated by measuring the relative electrolyte conductivity (REC) of leaves following the protocol described by Deshmukh et al. (1991). Then 0.2-g discs were briefly rinsed with deionized water and immersed in a test tube with 30 mL of deionized water for 12 h. The electrical conductivity (EC1) of the solution was measured with a conductivity meter (Model DJS-1C; Shanghai Analytical Instrument Co., Shanghai, China). Then the samples were heated at 100°C for 20 min and the conductivity (EC2) in the bathing solution was determined. Membrane permeability was calculated as the ratio of EC1/EC2.

### Determination of the superoxide anion (O^.−^_2_) production rate and the peroxide (H_2_O_2_) content

The O^.−^_2_ production rate was measured by monitoring the nitrite formation from hydroxylamine in the presence of O^.−^_2_ as described by Wang and Luo (1990). Frozen leaf tissue powder (0.2 g) from five plants was homogenized with 5 mL of 50 mM potassium phosphate buffer (pH 7.8). After a 15-min centrifugation at 4°C and 5000 × *g*, 1 mL of supernatant was mixed with 0.9 mL of 50 mM potassium phosphate buffer (pH 7.8) and 0.1 mL of 10 mM hydroxylamine hydrochloride and incubated for 30 min at 25°C. The incubated solution (1 mL) was added to 1 mL of 17 mM 3-aminobenzenesulfonic acid and 1 mL of 7 mM 1-naphthylamine, and then incubated for 20 min at 25°C. The absorbance was measured at 530 nm. A standard curve with NO^−^_2_ was used to calculate the O^−^_2_ production rate from the reaction equation of O^.−^_2_ with hydroxylamine. The O^.−^_2_ production rate was expressed as μm h^−1^ mg^−1^ protein.

Frozen leaf powder from five seedlings (0.2 g) was finely ground and homogenized with 20 mL of acetone at 0°C with the method described by Patterson et al. (1984) to determine H_2_O_2_ content. After a 15-min centrifugation at 6000 × *g* at 4°C, the supernatant was collected and 1 mL of supernatant was mixed with 0.1 mL of 5% titanium sulfate and 0.2 mL of ammonia and centrifuged for 10 min at 6000 × *g* at 4°C. The pellets were dissolved in 10% (v/v) H_2_SO_4_ (3 mL) and centrifuged for a further 10 min at 5000 × *g*. The absorbance of the supernatant was measured at 410 nm. The H_2_O_2_ content was calculated using H_2_O_2_ as a standard and expressed as mM mg^−1^ protein.

### Superoxide dismutase (SOD), catalase (CAT), and peroxidase (POD) activity

The leaf tissues were collected from one plant randomly selected from each replicate per treatment, cleaned with tissues to remove any surface contamination, and immediately frozen in liquid nitrogen and stored at −70°C. Then, 0.3 g of frozen leaves was ground in a mortar with 8 mL of 50 mM phosphate buffer solution (pH 7.8) containing 1% polyethylene pyrrole (PVP) at 4°C. The homogenate was centrifuged at 7500 × *g* for 15 min at 4°C. The supernatant was collected to indicate the activities of the enzymes.

SOD activity was assayed by monitoring its ability to inhibit the photochemical reduction of nitroblue tetrazolium (Beauchamp and Fridovich, 1971). One unit of SOD was defined as the amount of enzyme needed to inhibit the reduction of cytochrome c by 50%. It is expressed as U g^−1^ FW. CAT activity was measured by monitoring the disappearance of H_2_O_2_ (Díaz-Vivancos et al., 2008). This was detected by measuring the decrease in absorbance (at 240 nm) of a reaction mixture consisting of 1.5 mL of 50 mM sodium phosphate buffer (pH 7.8), 0.3 mL of 100 mM H_2_O_2_, and 0.2 mL of enzyme extract. One CAT unit was defined as the amount of enzyme needed to decompose 1 mmol H_2_O_2_ min^−1^ under these assay conditions. Specific CAT activity is given in U g^−1^ FW min^−1^. The POD activity in the leaves was estimated with a method described by Thomas et al. (1982) using guaiacol as the substrate. Specific POD activity is given in U g^−1^ FW min^−1^.

### Stomatal structure

The fourth leaf from the plant top was harvested from each plant randomly selected from each replicate per treatment. Portions of the epidermis were removed from the middle of the leaf using a razor blade, and immediately fixed in 2.5% (v/v) glutaraldehyde (0.1 mol L^−1^ phosphate buffer, pH 7.2) for at least 4 h. Then the samples were transferred to a mixture of ethanol and isoamylacetate (v:v = 1:1) for 30 min after dehydration in a graded series of ethanols. Finally, the slides were analyzed using a scanning electron microscope (TM-1000, Hitachi, Tokyo, Japan), and the single stomatal pore area per unit area was measured using Motic Images Plus 2.0 (Motic Ltd., Taiwan). Five images were analyzed per leaf. There were one leaf per plant and five plants per treatment (Snider et al., 2009).

### Chloroplast ultrastructure

To observe the chloroplast ultrastructure of the mesophyll cell, the leaves sampled for examination of the structure of the stoma (described above) were immediately fixed in 2.5% (v/v) glutaraldehyde (0.1 M phosphate buffer, pH 7.2) for at least 4 h after being cut from the plants. Then the samples were immersed in 1% (v/v) osmium acid for post-fixation and embedded in resin for ultrathin sectioning and examination with a transmission electron microscope (H7650, Hitachi) (Deng et al., 2012).

### Data analysis

Statistical analysis was conducted using a One-Way analysis of variance (ANOVA) with SPSS software version 16.0 (SPSS, Chicago, IL, U.S.), and Duncan's multiple range test was used to detect differences between means. The *P*-value was set at 0.05 and 0.01 for the ANOVA and Duncan's multiple range tests, respectively.

## Results

### Plant growth and development

**Figure 2:**
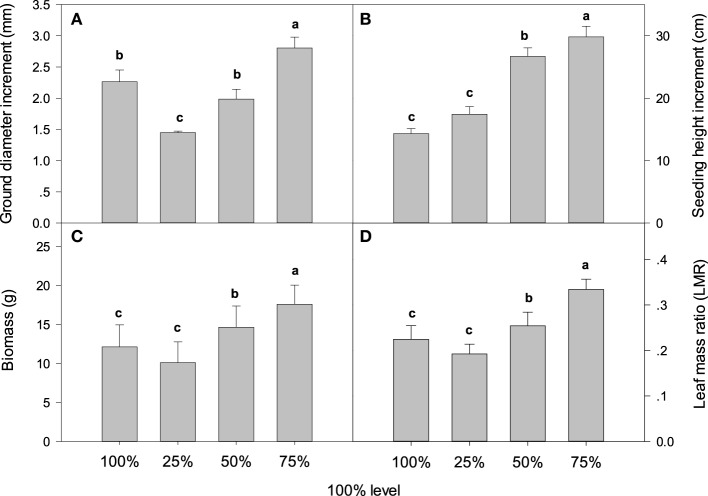
**Ground diameter increment (A), seedling height increment (B), biomass (C), and leaf mass ratio (LMR) (D) of *C. acuminata* in response to different levels of irradiance**. The values presented are the means ± *SE*. Different letters indicate significant differences between irradiance treatments (*P* < 0.05); *n* = 5. 100% level = 1500 ± 30 μmol m^−2^ s^−1^.

Clear external differences were observed in plants grown for 60 days under different light intensities. The size of the whole plant including the leaves grown under 75% irradiance was visibly larger than those in the other treatment groups (Figure 1). Compared with 100% irradiance, the LA increase was inaccordance with the increase of light intensity and 75% light irradiance resulted in the highest LA (Figure 1). Light intensity had different effects on the growth of *C. acuminata* seedlings: 75% light irradiance resulted in the highest total biomass, ground diameter, LMR, and seedling height. On the contrary, 25% irradiance resulted in the lowest total biomass, ground diameter, and LMR (Figure 2). Specifically, the total biomass, ground diameter, plant height, and LMR in seedlings under 75% irradiance were 45.1% (*P* < 0.05), 23.8% (*P* < 0.05), 108.3% (*P* < 0.05), and 32.7% (*P* < 0.05) higher, respectively, than those in plants grown under 100% light irradiance (Figure 2, PAR = 1500 ± 30 μmol m^−2^ s^−1^, control).

### Photosynthetic parameters

The effects of different light intensities on the leaf photosynthetic parameters are presented in Table 1. Compared with 100% light irradiance, plants exposed to 25% irradiance showed less leaf net photosynthesis (Pn) while 50% and 75% irradiance increased the Pn. The greatest leaf stomatal conductance (gs) was observed under 75% irradiance, and the lowest under 25% irradiance. When the light intensity decreased from 75% to 25% irradiance, a decrease in gs was observed. The transpiration rate (T) varied significantly with light intensity in *C*. *acuminata* seedlings. Compared with 100% light irradiance, 50% and 75% irradiance increased T by 22.12% (*P* < 0.05) and 25.2% (*P* < 0.05), respectively, whereas 25% irradiance decreased Tr by 4.1% (*P* > 0.05). Similar responses were observed in the intercellular [CO_2_] (Ci) and water use efficiency (WUE), i.e., the highest values were observed in plants under 75% irradiance and the lowest in those under 25% irradiance. No significant differences were observed between the 50% irradiance treatment and the control group (*P* > 0.05).

**Table 1:** **Net photosynthetic rate (Pn), stomatal conductance (gs), intercellular CO_2_ concentration (Ci), transpiration rate (T), and water use efficiency (WUE) of *C. acuminata* leaves were subjected to different levels of irradiance**.

**Treatment (100% level)**	**Photosynthetic parameters**				
	**Pn (μmol m^−2^ s^−1^)**	**gs (mmol m^−2^ s^−1^)**	**T (mmol m^−2^ s^−1^)**	**Ci (mmol mol^−1^)**	**WUE (%)**
100%	10.10±0.49c	0.22±0.01b	4.19±0.04bc	270.55±2.4b	2.06±0.11b
25%	8.13±0.68d	0.14±0.01c	4.02±0.06c	223.59±1.56c	1.76±0.10c
50%	11.79±0.49b	0.17±0.01c	5.12±0.06b	277.15±1.73b	2.11±0.06b
75%	14.35±0.56a	0.30±0.02a	5.25±0.25a	294.63±2.7a	2.66±0.05a

*The values presented are the means ± SE. Different letters indicate significant differences between irradiance treatments (P < 0.05); n = 5. 100% level = 1500 ± 30 μmol m^−2^ s^−1^*.

### Photosynthetic pigments and chlorophyll fluorescence

**Figure 3:**
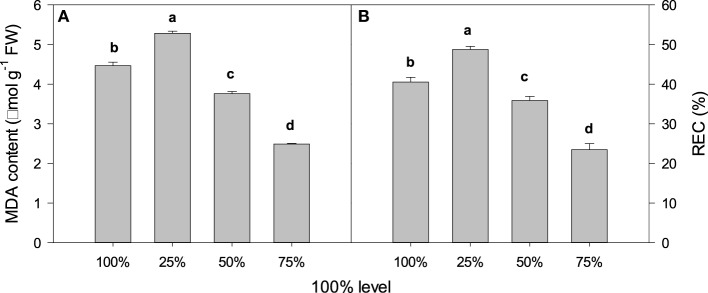
**Light intensity and (A) MDA content and (B) relative electrical conductivity (REC) in *C. acuminata* leaves**. The values presented are the means ± *SE*. Different letters indicate significant differences between irradiance treatments (*P* < 0.05); *n* = 5. 100% level = 1500 ± 30 μmol m^−2^ s^−1^.

Variations in the levels of photosynthetic pigments, including Chla, Chlb, and carotenoids (Car) were evaluated in *C. acuminata* seedlings under different levels of light (Figure 3). There were lower Chla, Chlb, and Car concentrations and smaller Chla/b ratios in shaded plants than in plants grown under full sunlight. Irradiance of 25%, 50%, and 75% resulted in lower Chla, Chlb, and Car levels and higher Chla/b ratios (Table 2). The lowest Chla, Chlb, and Car contents and the highest Chla/b ratio was recorded under 100% irradiance (Table 2).

**Table 2:** **Chlorophyll a (Chla), chlorophyll b (Chla), carotenoids (Car), Chl(a+b), chlorophyll a:b ratio (Chla/b) and Car/ Chl(a+b) in *C. acuminata* leaves in response to different levels of irradiance**.

**Treatment 100% level**	**Chla (mg/g)**	**Chlb (mg/g)**	**Car (mg/g)**	**Chl(a+b) (mg/g)**	**Chla/b**	**Car/Chl(a+b)**
100%	1.13±0.03c	0.35±0.01c	0.53±0.10c	1.66±0.05c	3.12±0.11a	0.52±0.003a
25%	2.31±0.14a	0.75±0.05a	0.94±0.03a	3.10±0.16a	2..43±0.04c	0.29±0.009c
50%	1.84±0.20b	0.58±0.08b	0.88±0.06a	2.40±0.05b	2.62±0.08b	0.30±0.004c
75%	1.54±0.24b	0.52±0.08b	0.72±0.01b	1.93±0.48c	2.83±0.13b	0.38±0.006b

*The values presented are the means ± SE. Different letters indicate significant differences between irradiance treatments (P < 0.05); n = 5. 100% level = 1500 ± 30 μmol m^−2^ s^−1^*.

The effects of light intensity on Fo, Fm, Fv/Fm, Φ PSII, NPQ, and qP in *C. acuminata* are shown in Table 3. The highest value of Fo was detected under 25% irradiance and the lowest under 75% irradiance. Irradiance at 25%, 50%, and 75% showed higher Fm values and the highest value was observed under 75% irradiance. The Fv/Fm, qP, and ΦPSII values were lower under 25% irradiance (*P* < 0.05) but were higher under 50% (*P* < 0.05) and 75% (*P* < 0.05) than those in controls. Specifically, compared with plants subjected to 100% irradiance, ΦPSII was increased by 15% (*P* < 0.05) and 37.5% (*P* < 0.05) under 50% and 75% irradiance, respectively, but was decreased by 10% (*P* < 0.05) under 25% irradiance. Similar tendency was observed in qP. However, no significant differences in qP were observed between the 50% irradiance treatment and the control groups (*P* > 0.05). In contrast, NPQ value showed a reduction of 17.6% (*P* < 0.05), 20.1% (*P* < 0.05), and 52.1% (*P*<0.05) under 25%, 50% and 75% irradiance, respectively, than those in controls.

**Table 3:** **Chlorophyll fluorescence parameters measured in *C. acuminata* in response to different light levels of irradiance**.

**Treatment (100% level)**	**Chlorophyll fluorescence parameters of *C. acuminata***					
	**Fo**	**Fm**	**Φ_PSII_**	**Fv/Fm**	**qP**	**NPQ**
100%	0.48±0.01ab	1.48±0.01d	0.40±0.01c	0.73±0.01c	0.73±0.01b	1.19±0.05a
25%	0.56±0.01a	1.64±0.02c	0.36±0.01d	0.70±0.01d	0.70±0.02c	0.98±0.01b
50%	0.40±0.01b	1.71±0.02b	0.46±0.01b	0.76±0.01b	0.79±0.01b	0.95±0.01b
75%	0.36±0.02c	1.93±0.02a	0.55±0.01a	0.82±0.01a	0.84±0.01a	0.57±0.02c

*The values presented are the means ± SE. Different letters indicate significant differences between irradiance treatments (P < 0.05); n = 5. 100% level = 1500 ± 30 μmol m^−2^ s^−1^*.

*Fo indicates initial fluorescence; Fm is the maximal fluorescence; Φ _PSII_ is actual photochemical efficiency of PSII, Fv/Fm is a ratio of variable fluorescence over the maximum fluorescence value, qP is photochemical quenching, and NPQ is non-photochemical quenching*.

### MDA content and membrane permeability, O^.−^_2_ production rate, H_2_O_2_ content, and POD, CAT, and SOD activities

Compared with the 100% light irradiance, 25% irradiance increased MDA content and membrane permeability, whilst 50% and 75% decreased MDA content and membrane permeability. Seedlings grown under 75% irradiance exhibited the lowest MDA content and membrane permeability (Figure 3).

**Figure 4:**
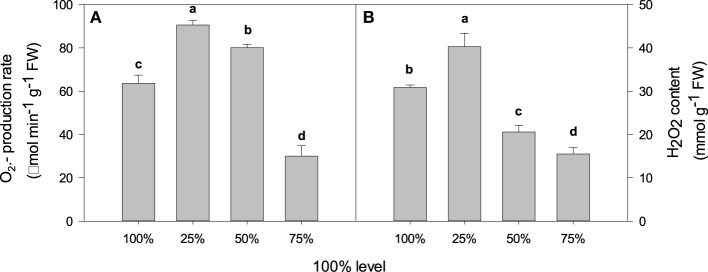
**Light intensity and the (A) O^.−^_2_ production rate and (B) and H_2_O_2_ content in *C. acuminata* leaves**. The values presented are the means ± *SE*. Different letters indicate significant differences between irradiance treatments (*P* < 0.05); *n* = 5. 100% level = 1500 ± 30 μmol m^−2^ s^−1^.

**Figure 5:**
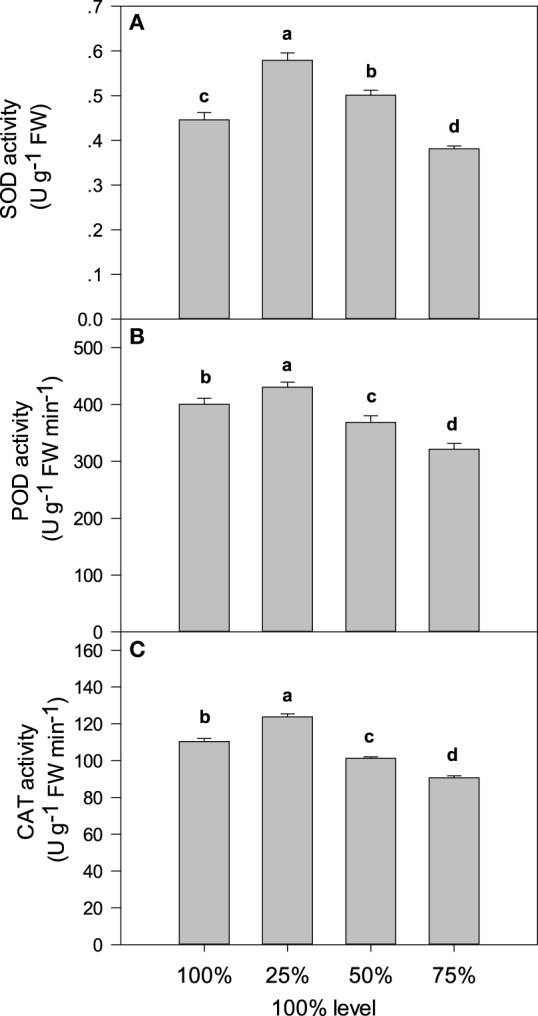
**Light intensity and (A) SOD, (B) POD, and (C) and CAT activity in *C. acuminata* leaves**. The values presented are the means ± *SE*. Different letters indicate significant differences between irradiance treatments (*P* < 0.05); *n* = 5. 100% level = 1500 ± 30 μmol m^−2^ s^−1^.

The O^.−^_2_ production rate was significantly higher under 25% and 50% irradiance than when in 100% light irradiance, by 42.29% (*P* < 0.05) and 25.78% (*P* < 0.05), respectively (**Figure 5A**). Conversely, 75% irradiance treatment was associated with 52.8% less O^.−^_2_ production than 100% light irradiance (*P* < 0.05) (Figure 4A). H_2_O_2_ content was 33.26% (*P* < 0.05) and 49.7% (*P* < 0.05) lower under 50% and 75% irradiance, respectively, but increased by 30.51% (*P* < 0.05) under 25% irradiance compared with the control (Figure 4B).

The levels of SOD, POD, and CAT activity in *C. acuminata* seedlings under different levels of light intensity are shown in Figure 5. Compared with 100% light irradiance, the 25% and 50% irradiance increased SOD activity by 90.1% (*P* < 0.05) and 27.0% (*P* < 0.05), respectively. However, 75% irradiance decreased SOD activity by17.3% (*P* < 0.05). Interestingly, there were similar profiles of activities of POD and CAT in different light-treated seedlings. Twenty-five and fifty percent irradiance increased POD and CAT activity significantly, whilst 75% decreased POD and CAT activity compared with 100% light irradiance.

### Stomatal structural traits

**Figure 6:**
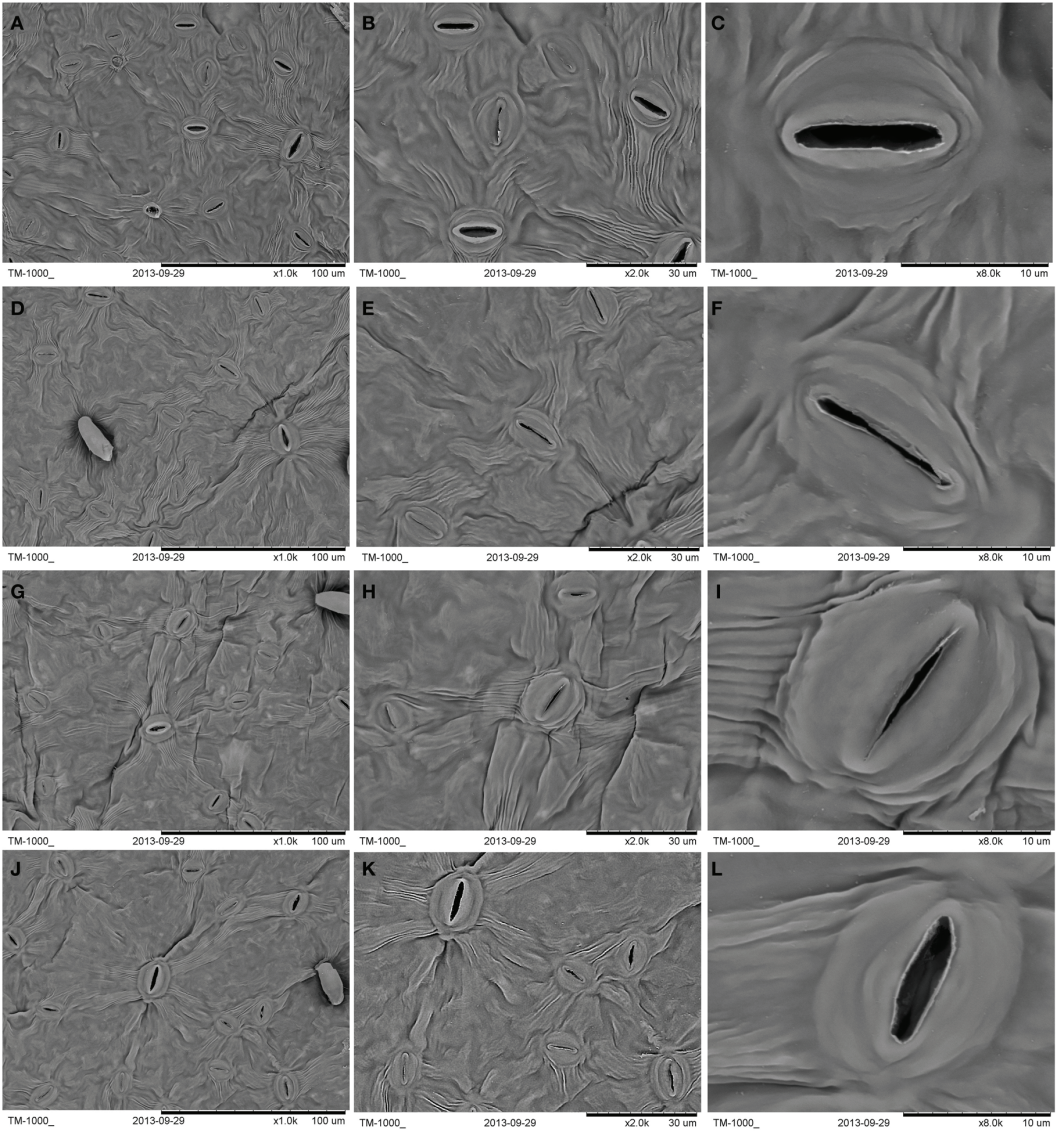
**Light intensity and stomatal traits in *C. acuminata* leaves under (A–C) 100% irradiance, (D–F) 25% irradiance, (G–I) 50%irradiance, and (J–L) 75% irradiance**. 100% level = 1500 ± 30 μmol m^−2^ s^−1^.

Different light intensities were associated with differences in the stomatal traits (Table 4, Figure 6). The number of stomata per unit LA was lower under 50% and 25% irradiance than in controls. The highest number of stomata per unit LA was observed in plants under 75% irradiance. When irradiance decreased from 100% to 75%, a gradual increase in the stomatal aperture was observed. The highest number of stomatal apertures was detected under 75% irradiance and the smallest under 25% irradiance (Table 4). Stomatal length was significantly (*P* < 0.05) higher under 75% irradiance and significantly (*P* < 0.05) lower under 25% irradiance than in controls. No significant difference was observed in the stomatal length between the 50% and 100% irradiance (Table 4). Stoma was wider in plants under 75% and 50% irradiance than in controls. However, there was no significant difference between the control and 25% irradiance (*P* > 0.05) (Table 4).

**Table 4:** **Light intensity and stomatal traits in *C. acuminata* leaves**.

**Treatment (100% level)**	**Stomatal traits of *C. acuminata***			
	**Stomatal**	**Stomatal**	**Stomatal**	**Stomatal**
	**length (μm)**	**width (μm)**	**number (m m^−2^)**	**aperture (μm)**
100%	11.56±0.62b	4.25±0.05c	12.27±0.63b	0.61±0.02b
25%	10.51±0.37c	4.41±0.18c	9.35±0.57c	0.38±0.02c
50%	11.89±0.60b	5.05±0.16b	11.02±0.60b	0.47±0.01bc
75%	13.67±0.60a	5.78±0.25a	16.34±0.57a	0.75±0.02a

*The values presented are the means ± *SE*. Different letters indicate significant differences between irradiance treatments (P < 0.05); n=5. 100% level=1500 ± 30 μmol m^−2^ s^−1^*.

### Chloroplast ultrastructure

**Figure 7:**
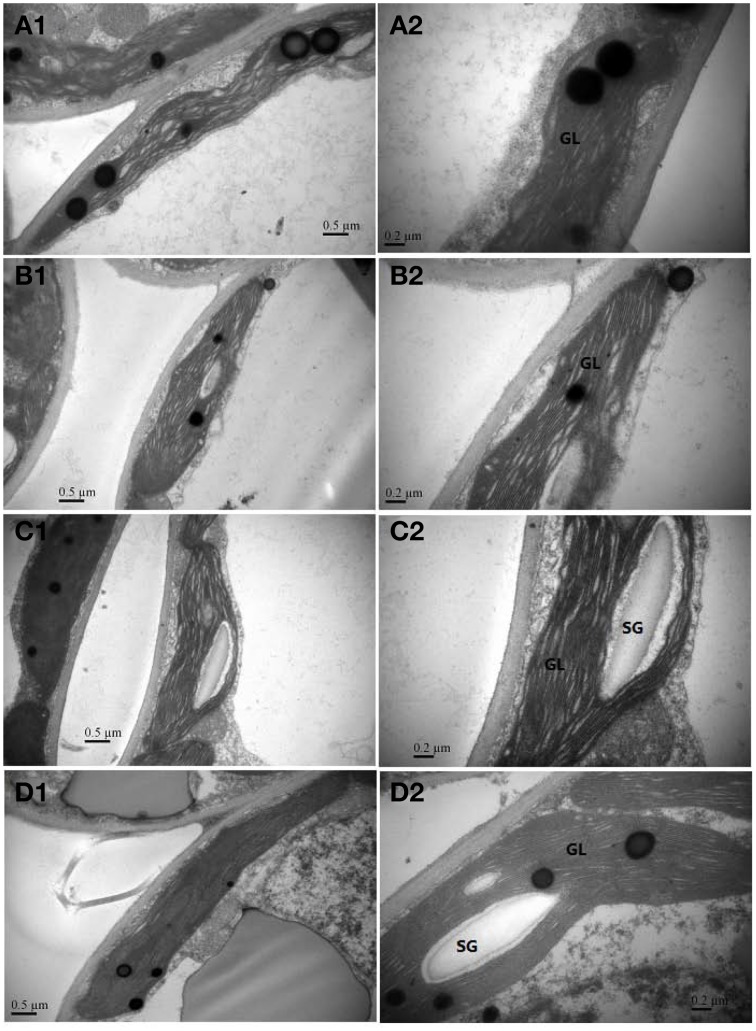
**Chloroplast ultrastructure of *C. acuminata* leaves under (A1,A2) 100% irradiance, (B1,B2) 25% irradiance, (C1,C2) 50%irradiance, and (D1,D2) 75% irradiance**. Abbreviation: SG, starch grains; GL, grana lamellae. 100% level = 1500 ± 30 μmol m^−2^ s^−1^.

Light intensity had a marked influence on the size, shape, and number of chloroplasts in *C. acuminata* seedlings (Table 5 and Figure 7). The number of grana was 54.3% (*P* < 0.05) and 44.1% (*P* < 0.05) higher under 75% and 50% irradiance, respectively, than that in controls. It was 11.1% (*P* < 0.05) lower under 25% irradiance than in controls. There were 24.1% and 10.4% fewer grana lamellae per plant under 25% and 50% irradiance, respectively, than under 100% (*P* < 0.05). There were 24.0% more under 75% irradiance than in controls (*P* < 0.05). Furthermore, 75% irradiance increased the thickness of the grana, whilst 25% irradiance decreased the thickness of the grana compared with the control. Interestingly, no significant difference was observed in the thickness of grana between the control and 50% irradiance (*P* > 0.05).

**Table 5:** **Light intensity and chloroplast ultrastructure of *C. acuminata* leaves**.

**Treatment (100% level)**	**Chloroplast ultrastructure of *C. acuminata***				
	**Chloroplast length (μm)**	**Chloroplast width (μm)**	**Grana number**	**Grana thickness (μm)**	**Number of grana lamellae**
100%	3.89±0.02c	2.09±0.09c	8.76±0.37c	0.020±0.0006b	12.01±0.61b
25%	4.07±0.03c	3.73±0.01a	7.89±0.33d	0.018±0.0005c	9.12±0.39d
50%	5.74±0.07a	1.77±0.14d	11.26±0.38b	0.025±0.0004b	10.76±0.41c
75%	4.75±0.08b	2.39±0.07b	13.57±0.41a	0.034±0.0001a	14.89±0.62a

*The values presented are the means ± SE. Different letters indicate significant differences between irradiance treatments (P < 0.05); n = 5. 100% level = 1500 ± 30 μmol m^−2^ s^−1^*.

## Discussion

*C. acuminata* tree has been found to have a higher level of CPT which has been used as a drug for cancer treatment (Liu et al., 1997). It is generally recognized that light intensity plays an important role in plant growth, morphology, photosynthetic capacity, various aspects of physiology and biochemistry (Gottschalk, 1994). Meanwhile the light intensity could affect secondary metabolites (Liu et al., 1997; Müller et al., 2013). As one kind of secondary metabolites, CPT production not only depends on leaf biomass production but also concentration of CPT in leaf. This study may improve the understanding of the effect of light intensity on the growth and physiology of this species for standardized cultivation to optimize plant growth. Current results clearly suggest that optimal light intensity can improve the biomass of *C. acuminata* by enhancing photosynthesis, reducing ROS accumulation and maintaining stomata and chloroplast structure.

Light intensity affects a range of plant characteristics, such as height, total biomass, and leaf size (Dai et al., 2009). In the current study, *C*. *acuminata* seedlings grown under 75% irradiance performed the best (Figures 1, 2), indicating that 75% light intensity was the optimum for the growth. Insufficient light may stress plants by limiting photosynthesis, resulting in reduced net carbon gain and plant growth. Conversely, high light levels may damage the photosynthetic apparatus (Larcher, 1995). In our study, we also found that shorter seedling height and less total biomass were detected in plants grown under 25% and 100% irradiance (Figures 1, 2). These findings are similar to the results reported by Zhao et al. (2012) for herbaceous peony. Furthermore, the highest Pn, T, gs, and Ci were observed in plants under 75% irradiance (Table 1). The decreases in Pn associated with the reduced T, gs, and Ci under 25%, 50%, and 100% irradiance indicated that stomatal limitation occurred, which was consistent with lower values in stomatal length, width, apertures, and number of stoma per unit LA (Table 4). Similar result was also reported in blueberry tree (Guo et al., 2006). The lower stomatal number and aperture per unit LA help reduce the burden of the photosynthetic reaction center and protect plants from light deficiency (Lawson et al., 2011).

Leaf chlorophyll content is another important factor determining the rate of photosynthesis and plant growth. In this study, the decreases in Chl a and b contents were observed under 100% irradiance (Table 2), suggesting excessive-irradiance-induced damage to pigments (Shao et al., 2014). This finding was consistent with the response of *T. hemsleyanum* (Dai et al., 2009). In addition, the reduction in the Chla/b ratio was observed under shade treatments mainly due to the increase in Chlb content (Table 2) that might contribute to the enhancement of the light capture for photosynthesis (Murchie and Horton, 1997). Interestingly, the highest concentrations of total Chl a+b and the lowest Pn and Chla/b ratio were observed under 25% irradiance among the four light treatments (Table 2). Murchie and Horton (1997) suggested one strategy for long-term acclimation was due to secondly changes in chlorophyll a/b ratio and P_max_ indicative of alterations at the chloroplast level, which were not associated with a change in chlorophyll content per unit LA. Similarly, changes in Chla/b ratio and Pn might considered be as a response to different light conditions.

Chlorophyll fluorescence serves as one of the most powerful tools in the assessment of photosynthetic performance in plant physiological experiments and has also been used as a non-invasive method to examine photochemical processes in intact leaves at high light intensities (Schreiber et al., 1995). In the current study, Fm, Fv/Fm, and ΦPSII values were higher under 75% irradiance than those under other treatments (Table 3), which suggests that 75% irradiance increased the efficiency of photosynthesis (Tang et al., 2015). The highest qP value was recorded in *C. acuminata* seedlings under 75% irradiance (Table 3), which contributes to the separation of electric charges in the reaction center, electron transport, and PSII yield (Mao et al., 2007). The lowest NPQ value accompanied with the highest ΦPSII in the plants under 75% irradiance indicated efficient utilization of the energy absorbed by the antenna pigments in PSII (Maxwell and Johnson, 2000; Dai et al., 2009). However, the highest NPQ value and Car/Chl ratio NPQ under 100% irradiance suggested that non-photochemical dissipation of excessive light energy was as the main pathway. Generally, plants subjected to stress typically have lower Fv/Fm values than non-stressed plants (Björkman and Demmig, 1987; Baker, 2008). In the present study, Fv/Fm was significantly reduced accompanied with decreased Pn under 100% irradiance compared with 75% light intensity (Tables 1, 3), indicating the occurrence of chronic photoinhibition under such circumstances. Excessive light energy damages the reaction center and inhibits PSII electron transport and ROS can be over-produced by the direct transfer of excitation energy from chlorophyll, leading to the damages of the membrane and lipid peroxidation (Gill and Tuteja, 2010).

The MDA content typically reflects the level of lipid peroxidation in biomembranes, including the peroxidative degradation of thylakoid lipids. The REC is a direct indicator of the level of membrane permeability. In this study, compared with 75% irradiance, 25% and 100% irradiances significantly increased MDA content and REC value (Figure 3), which indicated a high level of oxidative damage to lipid membranes. Lipid peroxidation and membrane damage are typically caused by the formation of ROS, such as O^.−^_2_, hydroxyl ions (OH) and H_2_O_2_ (Krause, 1988). In the present study, compared with 75% irradiance, plants under 100% irradiance had higher O^.−^_2_ production rates and H_2_O_2_ levels associated with higher antioxidant enzyme activity (POD, SOD, and CAT) (Figures 4, 5) that indicated the enhancement of scavenging ability of ROS. This result was consistent with the report on olive trees by Sofo et al. (Sofo et al., 2004). Similar results were also observed under 25% irradiance (Figures 4, 5), which may partly be explained by the lower energy utilizing capacity, as the 25% irradiance-treated plants had lower Pn than the 75% irradiance-treated plants (Zhao et al., 2012).

Chloroplasts are the only organelles in which photosynthesis occurs, and the photoreactions are localized in the internal chloroplast membrane (i.e., the thylakoid). The structure and integrity of the thylakoids is critical to effective photosynthesis (Liu et al., 2012). In this study, leaves grown under 75% irradiance had better-developed grana and contained more thylakoids than those in the other treatments (Figure 7), which was consistent with the highest photosynthetic rates and total biomass at 75% irradiance (Table 1; Figure 2). Compared with 75% irradiance, plants under 100% and 25% irradiances exhibited the increases in MDA content, membrane permeability, ROS contents (O^.−^_2_ production rates and H_2_O_2_ levels) (Figures 3, 4). The swollen chloroplast and irregular shape of the grana with decreased lamellae and disorganization of thylakoid membrane system were observed under excessive and insufficient light conditions. The reason might be that lipid peroxidation and ROS accumulation caused the loss of membrane integrity, thereby leading to irregularly shaped chloroplasts. Similar results were reported by Tang et al. (2015) who found that high/low light intensities accelerated the damage of chloroplasts.

In summary, plant growth was the strongest under 75% irradiance, which was attributable to the increase of photosynthesis, the reduction in the accumulation of ROS, the maintenance of the stomatal and chloroplast structure. These responses were associated with improvements in the photochemical efficiency of PSII. Plants subjected to full light conditions suffered photoinhibition caused by exposure to excessive light, and plants grown under 25% irradiance suffered from light deficiency. This study provides a better understanding of the responses of physiological and biochemical mechanisms to light intensity in *C. acuminata* seedlings. This serves a theoretical basis for the standardized cultivation of *C. acuminata* seedlings.

### Conflict of interest statement

The authors declare that the research was conducted in the absence of any commercial or financial relationships that could be construed as a potential conflict of interest.
